# Nanodrug delivery systems targeting ferroptosis as an innovative therapeutic approach for Rheumatoid Arthritis

**DOI:** 10.1016/j.mtbio.2025.101804

**Published:** 2025-04-24

**Authors:** Xiaolin Dai, Yu Zheng, Jianrong Cui, Yuqi Zeng, Bo Yang, Zhanlin Zhang

**Affiliations:** a Department of Pharmacy, Chengdu Seventh People's Hospital (Affiliated Cancer Hospital of Chengdu Medical College), Chengdu, China; b Irradiation Preservation and Effect Key Laboratory of Sichuan Province, School of Bioscience and Technology, Chengdu Medical College, Chengdu, 610500, PR China; c The Second Affiliated Hospital of Chengdu Medical College, China National Nuclear Corporation 416 Hospital, Chengdu, 610051, PR China; d Department of Endocrinology and Metabolism, The Affiliated Hospital of Southwest Medical University, Luzhou, 646000, PR China; e Sichuan Clinical Research Center for Nephropathy, Luzhou, 646000, PR China; f Metabolic Vascular Disease Key Laboratory of Sichuan Province, Luzhou, 646000, PR China; g Sichuan-Chongqing Joint Key Laboratory of Metabolic Vascular Diseases, Luzhou, 646000, PR China

**Keywords:** Rheumatoid Arthritis, Ferroptosis, Nanodrug delivery systems, Lipid peroxidation, Reactive oxygen species

## Abstract

Rheumatoid Arthritis (RA) is a chronic inflammatory disease characterized by joint inflammation, progressive cartilage degradation, and bone erosion. Recent research has implicated ferroptosis not only in autoimmune hepatitis but also in the pathogenesis and progression of autoimmune disorders like RA. Consequently, numerous therapeutic strategies have begun to target the ferroptosis pathway, particularly in the design and development of nanodrug delivery systems (NDDSs). While previous reviews have comprehensively discussed the mechanisms of ferroptosis, related signaling pathways, and NDDS materials, recent studies have further elucidated the interplay between ferroptosis and various metabolic pathways, providing a robust theoretical basis for the design of NDDS-based ferroptosis strategies. This review focuses on investigating the role of ferroptosis in the development of RA, aiming to elucidate how targeting ferroptosis can offer novel therapeutic concepts and potential treatments for RA patients. Specifically, it summarizes the design strategies of ferroptosis-based NDDSs *via* different pathways and highlights the feasibility of RA treatment regimens based on the ferroptosis mechanism. Furthermore, the review critically discusses the current limitations of NDDSs and offers perspectives on future research directions in this field.

## Highlights

• Ferroptosis is involved in autoimmune hepatitis and contributes to the development of autoimmune disorders.
• Nanodrug delivery systems use nanomaterials' characteristics to enhance drug properties and achieve precise drug delivery.
• Nanodrug delivery systems targeting ferroptosis serve as a novel treatment approach to Rheumatoid Arthritis.

## Graphical abstract

**Figure:**
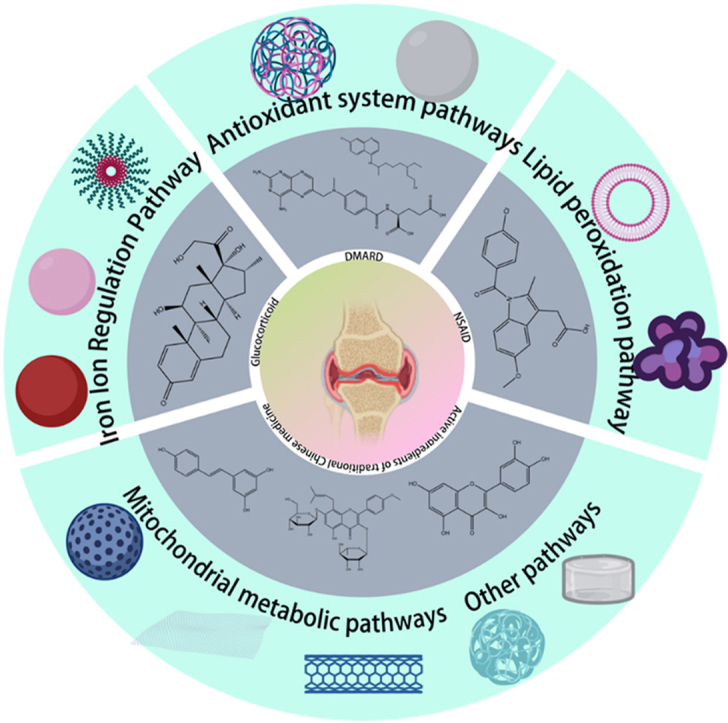

## 1 Introduction

Rheumatoid Arthritis (RA) is a complex autoimmune disorder with incompletely understood etiologies. Its hallmark clinical presentation is erosive, symmetrical arthritis, reflecting the progressive destruction of joint structures, typically affecting both sides of the body symmetrically [1]. RA is characterized by inflammatory changes within synovial joints, cartilage, synovial tissues, and extra-articular sites, extending beyond the joints to encompass surrounding tissues and organs. These pathological alterations ultimately result in irreversible joint deformity and functional impairment, significantly diminishing patients’ quality of life [2]. Globally, the prevalence of RA is approximately 1%, a relatively high figure, underscoring its widespread impact. Regarding gender distribution, RA prevalence is notably higher in females than in males, with a ratio of approximately 2:1 to 3:1, suggesting increased susceptibility in females [3]. While RA can manifest at any age, the peak incidence occurs primarily around the age of 60, potentially linked to age-related changes in immune function. Importantly, studies have also confirmed that RA patients exhibit a significantly elevated risk of cardiovascular disease [4]. This increased risk may be attributed to the systemic inflammatory response associated with RA and potential adverse effects of certain RA medications. Cardiovascular disease frequently represents the leading cause of mortality in RA patients, further emphasizing the profound impact of RA on overall health and the necessity of a holistic approach to patient care, considering their multifaceted health needs [5].

The primary causative factors of RA remain to be fully elucidated; however, research has established its association with multiple factors, including genetic predisposition, environmental influences, lifestyle choices, inflammatory cytokines, and infectious agents [6]. These factors can interact to collectively promote the onset and progression of RA. Currently, the standard clinical treatment for RA primarily involves non-steroidal anti-inflammatory drugs, glucocorticoids, and disease-modifying anti-rheumatic drugs [7]. These medications can be further classified into conventional, biological, and targeted synthetic therapies [8]. While these treatments effectively alleviate RA symptoms and potentially slow disease progression, they have yet to provide a complete cure [9]. Therefore, it is crucial to investigate and develop novel therapeutics that target new pathways or employ innovative mechanisms of action to address the unmet clinical needs of RA [10]. Ferroptosis, a recently identified form of cell death, has been implicated in various diseases, including musculoskeletal disorders, inflammatory disorders, autoimmune disorders, Parkinson's disease, and cancer. In cells, ferroptosis is characterized by lipid peroxidation initiated by the accumulation of iron-dependent reactive oxygen species (ROS) [11]. Iron, essential for normal cellular function in mammalian systems, plays a significant role in maintaining joint health. Excess iron can generate substantial amounts of harmful ROS through the Fenton or Haber-Weiss reactions [12], leading to cellular damage to DNA, proteins, and lipids, ultimately resulting in cell death. Notably, research over the past few decades has demonstrated that some RA patients exhibit abnormally high iron levels in their joints [13]. Maintaining iron homeostasis at both the systemic and cellular levels necessitate strict regulation of iron acquisition, storage, and excretion. Emerging evidence strongly suggests a correlation between ferroptosis and other clinical manifestations of RA. Consequently, investigating the involvement of ferroptosis in RA pathogenesis and developing innovative therapeutic strategies that specifically target the ferroptosis pathway hold significant promise for improving the prognosis of RA patients. However, enhancing the specificity of ferroptosis activators to minimize off-target effects remains a critical challenge for clinical application. Nanotechnology offers novel opportunities for targeted ferroptosis modulation in RA therapy [14]. Nanoparticle drug delivery systems (NDDSs) are crucial in medicine, particularly for cancer and RA. By improving drug solubility, bioavailability, and *in vivo* retention while preventing premature degradation, NDDSs facilitate targeted delivery to cells or organelles, reducing side effects and enhancing efficacy [15,16]. In RA, traditional drug delivery faces limitations and toxicity, but nanotechnology offers solutions: biocompatible PLGA nanoparticles or surface-modified carriers (*e.g.*, 2C5 antibody-targeted NETs) improve precision [[17], [18], [19]]. NDDS can deliver small molecules (*e.g.*, methotrexate), proteins, genes, or cGAS-STING agonists, improving cancer therapy efficacy/toxicity ratios and targeting inflamed RA synovium to minimize systemic effects. They also modulate cancer and autoimmunity *via* immunotherapy [[20], [21], [22], [23]]. Existing reviews on RA primarily concentrate on the design of material-dependent NDDSs. This review aims to offer a concise overview of the development of NDDSs for modulating diverse mechanisms and targets within ferroptosis-based RA remission therapy.

## 2 The specific mechanisms of ferroptosis in RA

**Fig. 1:**
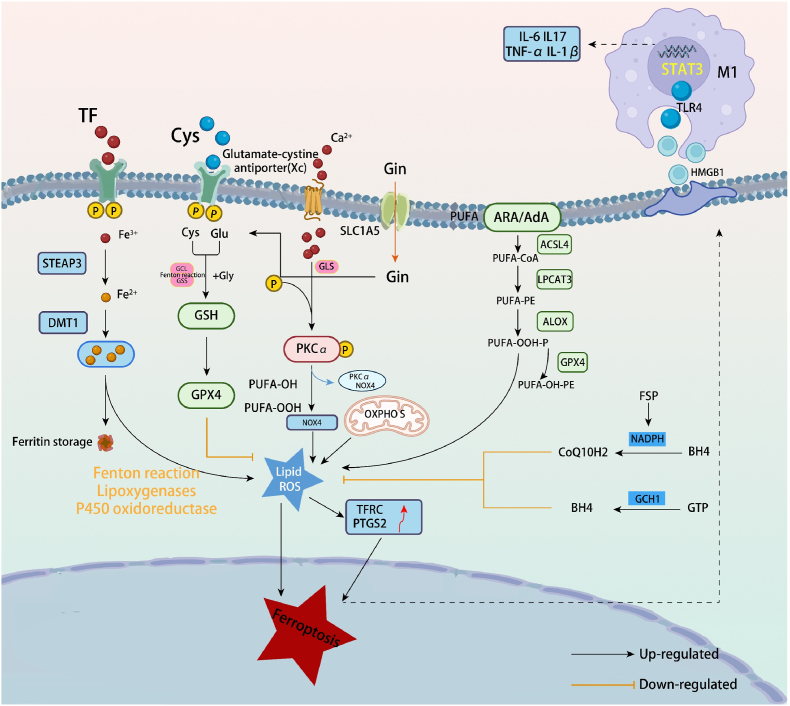
Overview of the mechanisms for efficient ferroptosis-based RA therapy.

Recent studies have demonstrated a strong link between ferroptosis and the pathogenesis of RA, opening new therapeutic possibilities [24]. While the process of ferroptosis is complex and not fully understood, it is known to involve alterations in cellular metabolism, including iron, lipid, and amino acid metabolism, among other key factors (Fig. 1).

### 2.1 Iron accumulation

Under physiological conditions, cellular iron homeostasis is maintained through a dynamic balance of uptake, storage, and release to support normal metabolic functions [25]. However, dysregulation of iron metabolism pathways (*e.g.*, increased absorption or impaired storage/export) may lead to free iron overload, triggering oxidative stress *via* the Fenton reaction and inducing ferroptosis [26,27]. In biological systems, iron exists as Fe^2+^ and Fe^3+^, with excess Fe^3+^ being sequestered into storage ferritin complexes through ferritin light chain (FTL), ferritin heavy chain (FTH1), and mitochondrial ferritin (MtFt) [28]. Cellular iron uptake occurs *via* transferrin receptor-mediated endocytosis, followed by transport to the labile iron pool (LIP) through ferroportin (FPN) and divalent metal transporter 1 (DMT1). Iron can be exported *via* the prominin-2-mediated multivesicular body pathway or stored in lysosomes [[29], [30], [31]]. Notably, iron serves as a cofactor for arachidonate lipoxygenases (ALOXs) and cytochrome P450-mediated lipid peroxidation, directly driving ferroptosis [32]. In RA, synovial iron deposition was first observed in 1968 [33], with subsequent studies demonstrating iron's multifaceted role in disease progression: iron dextran induces erythrocyte lipid peroxidation, depletes glutathione (GSH), and exacerbates synovitis [34]; excess iron promotes osteoclast differentiation *via* ROS while inhibiting osteoblast proliferation [35]; and concurrent activation of p38 MAPK with suppression of PI3K/Akt and JAK/STAT3 pathways triggers death of osteoprogenitor cells like MC3T3-E1 [36]. Clinical studies reveal elevated serum soluble transferrin receptor (sTfR) levels positively correlating with inflammatory markers in RA patients, despite paradoxically low systemic iron levels, suggesting systemic iron redistribution [37]. Synovial fluid analyses demonstrate significant increases in oxidative stress markers (8-OHdG, 4-HNE, malondialdehyde) in both RA patients and collagen-induced arthritis (CIA) mice [38]. Importantly, FTH1, FTL, and transferrin receptor expression are detected in synovial fibroblasts (FLS) and macrophages, with pro-inflammatory cytokines (Interleukin-1:IL-1, Interleukin-6: IL-6, Tumor Necrosis Factor-α: TNF-α) enhancing their iron-uptake capacity [39]. Recent studies highlight ferritinophagy's critical role in maintaining synovial proliferative regeneration, while glycine modulates GPX4 promoter methylation *via* S-adenosylmethionine levels, revealing novel therapeutic targets [40]. Although ferroptosis is increasingly recognized as an iron-dependent programmed cell death mechanism in RA, the precise mechanisms underlying macrophage survival abnormalities require further investigation [41,42].

### 2.2 Oxidative stress and lipid metabolism

Oxidative stress plays a pivotal role in the pathogenesis and progression of RA. ROS, generated during oxidative stress, contribute substantially to RA pathophysiology. Elevated ROS levels in the joint cavity of RA patients not only promote disease progression but also serve as key mediators of ferroptosis [43,44]. The observed depletion of vitamin C and vitamin E in RA patients’ blood further indicates systemic antioxidant deficiency [45]. Extensive research has elucidated the multifaceted effects of ROS in RA, including: (1) activation of matrix metalloproteinases (MMPs), (2) suppression of cartilage proteoglycan synthesis, (3) stimulation of FLS proliferation, and (4) induction of chondrocyte apoptosis [46,47]. These collective mechanisms ultimately lead to cartilage degradation and bone erosion. Moreover, oxidative stress disrupts T-cell homeostasis, aggravating immune dysregulation and accelerating RA progression [48]. Lipid peroxidation represents another critical pathological feature of RA. Reduced glutathione peroxidase (GPX) activity in RA patients impairs cellular defense against lipid peroxidation, thereby promoting ferroptosis [45]. Lipidomic analyses reveal significant alterations in plasma lipid metabolism, with marked elevations in lipid peroxidation products, underscoring the interplay between ferroptosis, oxidative stress, and lipid peroxidation in RA [49]. Furthermore, ferroptosis triggers innate immune responses, generating inflammatory mediators that perpetuate synovitis. Synovial tissue dysfunction and metabolic reprogramming drive the production of pro-inflammatory cytokines (*e.g.*, IL-1β, TNF-α, IL-6), which upregulate ferritin synthesis [50,51]. The consequent iron dyshomeostasis exacerbates synovial inflammation, culminating in irreversible joint damage.

### 2.3 The interplay between the immune microenvironment and ferroptosis

Ferroptosis represents a unique form of regulated cell death that exerts significant influence on both cellular termination and subsequent physiological and pathological cascades. This process activates innate immune responses and releases key inflammatory mediators, including IL-1β, TNF-α, and IL-6, through complex molecular mechanisms [52]. Immune infiltration analysis in RA revealed that genes such as PTGS2 and ENO1 are significantly associated with macrophage and T-cell infiltration. Pro-inflammatory factors (*e.g.*, IL-1β, TNF-α) released during ferroptosis activate the NF-κB and p38/JNK signaling pathways, thereby forming an inflammation-oxidative stress positive feedback loop that exacerbates synovitis [53]. Elevated iron levels may further exacerbate synovial inflammation, accelerate joint destruction, and ultimately impair joint function. Mitochondria, as cellular energy hubs, play a central role in RA inflammation [54]. Synovial mitochondrial dysfunction not only compromises cellular energy supply but also promotes the production of inflammatory mediators and ROS [45]. These mitochondrial-derived ROS activate critical signaling pathways, including NF-κB, p38 MAPK, and JNK, forming a self-reinforcing cycle with TNF-α that progressively worsens RA pathology [55]. Notably, Feldmann et al. identified TNF-α as a pivotal mediator in RA pathogenesis [56]. TNF-α activates NF-κB signaling to stimulate intracellular GSH production, which protects fibroblasts from lipid peroxidation stress and ferroptosis [57]. While this mechanism preserves fibroblast viability, it may paradoxically perpetuate synovial inflammation and cartilage degradation [58]. Supporting this hypothesis, Wu et al. demonstrated that etanercept potentiates the cytotoxic effects of the ferroptosis inducer imidazole ketone erastin (IKE) on fibroblasts [31]. This finding suggests that TNF-α inhibition sensitizes fibroblasts to ferroptosis, potentially mitigating joint inflammation and structural damage. These insights not only advance our understanding of RA pathogenesis but also reveal novel therapeutic opportunities. Combined targeting of TNF-α and ferroptosis pathways may represent a promising strategy for RA management. Synovial fluid analyses reveal differential lipid peroxidation levels in monocytes and macrophages, implicating ferroptosis in RA progression [55]. Macrophage ferroptosis contributes to RA initiation and development, with M2 macrophages exhibiting greater susceptibility than M1 macrophages [56]. This disparity stems from distinct P62/SQSTM1-GPX4 autophagic degradation patterns under iron overload conditions [57]. Such heterogeneity in ferroptosis susceptibility may underlie immune dysregulation in RA joints, with macrophages emerging as primary ferroptosis targets in RA lesions [58]. These findings provide new perspectives on RA pathology and identify potential therapeutic targets for addressing inflammatory and immune imbalances in RA.

### 2.4 Crosstalk between ferroptosis and other cell death mechanisms

In RA, multiple regulated cell death pathways-particularly ferroptosis, apoptosis, and necroptosis-exhibit complex interplay that collectively drives disease progression. The hyperproliferation of FLS in RA synovial tissue significantly contributes to both synovial inflammation and joint destruction. While ferroptosis mitigates inflammation by eliminating FLS, apoptosis serves to remove damaged cells and preserve tissue homeostasis. Conversely, necroptosis exacerbates joint pathology through synovial cell necrosis and subsequent release of pro-inflammatory mediators. Emerging evidence reveals bidirectional regulation between ferroptosis and apoptosis: ferroptosis-derived oxidative stress and lipid peroxidation products can activate apoptotic signaling pathways, while dysregulated apoptotic protein expression may compromise cellular antioxidant capacity, thereby exacerbating ferroptosis. Furthermore, necroptosis-associated inflammatory factors may enhance ferroptosis susceptibility, whereas ferroptosis-generated oxidative stress could reciprocally promote necroptotic cell death. This multifaceted crosstalk creates a vicious cycle that amplifies RA pathogenesis. Therapeutic modulation of these interconnected cell death pathways represents a promising strategy for RA management. Potential approaches include: (1) targeted regulation of ferroptosis-related protein expression or activity, (2) combination therapy utilizing ferroptosis modulators with conventional RA drugs to achieve synergistic therapeutic effects. Additionally, ferroptosis-associated molecular signatures show potential as diagnostic and prognostic biomarkers for RA, facilitating early detection, disease monitoring, and treatment response evaluation [59].

## 3 Clinical & experimental ferroptosis-based drugs for RA

**Fig. 2:**
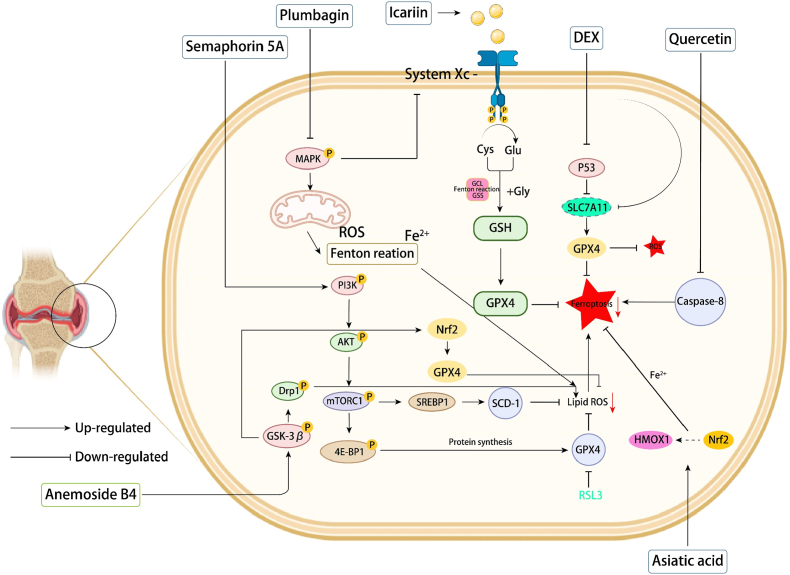
Some clinical drugs and experimental compounds for efficient ferroptosis-based RA therapy.

Current therapeutic interventions for RA exhibit diverse mechanisms of action involving ferroptosis modulation (Fig. 2). Glucocorticoids, particularly dexamethasone, promote ferroptosis by activating specific cellular pathways and upregulating dipeptidase-1 expression, consequently depleting GSH levels and enhancing cellular ferroptosis susceptibility [60]. Prednisolone demonstrates comparable ferroptosis-inducing effects. Among non-steroidal anti-inflammatory drugs (NSAIDs), indomethacin modulates malondialdehyde and GPX levels in sodium urate crystal-induced models, while ibuprofen suppresses GPX4 and solute carrier family 7 member 11 (SLC7A11) expression through inhibition of the nuclear factor erythroid 2-related factor 2 (Nrf2) pathway, thereby inducing ferroptosis in glioblastoma cells [61]. Conventional disease-modifying antirheumatic drugs (DMARDs) also influence ferroptosis pathways. Methotrexate-treated cells display characteristic ferroptosis markers including iron overload and ROS accumulation, while sulfasalazine has been shown to induce ferroptosis in neuroglioma cells [62]. Furthermore, hydroxychloroquine counteracts lipopolysaccharide-induced ferroptosis-related molecular alterations in monocytes, and cyclophosphamide promotes ferroptosis through enzyme-mediated ROS overproduction [63].

Recently, Zhao et al. has found that TNF-α can promote the synthesis of GSH in cells by activating NF-κB, thereby protecting fibroblasts from lipid peroxidation stress and resisting ferroptosis [64]. The TNF-α antagonist etanercept can significantly enhance the killing effect of low-dose ferroptosis inducer IKE on fibroblasts, thereby alleviating joint inflammation, preventing cartilage destruction, and inhibiting the progression of RA. Iron balance is essential for maintaining good health, and a deficiency of hepcidin can result in excessive iron accumulation in hereditary hemochromatosis and iron-loading anemia. This study utilized a functional screen to uncover iron regulators in human liver Huh7 cells [65]. The study discovered that the anti-RA drug auranofin can significantly increase the expression of hepcidin. Notably, essential signaling networks like as the bone morphogenetic protein/SMAD and IL-6/JAK2/STAT3 pathways are involved in regulating iron and are crucial for mediating the effects of auranofin. Moreover, auranofin can activate IL-6 *via* the NF-κB pathway. Acute treatment with 5 mg/kg auranofin in C57BL/6J mice can lower serum iron and transferrin saturation while activating the IL-6/hepcidin signal in the liver. Male Hfe−/− mice can be treated with 5 mg/kg auranofin for an extended period of time to activate IL-6/hepcidin signal in the liver and reduce systemic iron overload, female mice do not respond as well to this treatment. Additional investigation reveals that estrogen diminishes the capacity of auranofin to elicit IL-6/hepcidin signal in Huh7 cells, offering a molecular rationale for auranofin's ineffectiveness in female Hfe−/− mice. It is important to remember that excessive doses of auranofin might decrease thioredoxin reductase activity, which can lead to ferroptosis and lipid peroxidation through regulation of TXNRD. Thus, ferroptosis inhibitor ferrostatin does not contain its advantageous effects on iron metabolism, but it can provide substantial protection against liver damage caused by high-dose auranofin [66]. Ferroptosis is primarily regulated by TXNRD, but auranofin is a new activator that can trigger ferroptosis and hepcidin *via* separate pathways. This indicates that auranofin has potential application value in treating diseases related to hemochromatosis and hepcidin deficiency [66]. Additionally, active ingredients in traditional Chinese medicine, such as resveratrol, icariin, quercetin, baicalein, and asiatic acid, have also demonstrated the ability to affect the ferroptosis process through different pathways, providing new perspectives and potential therapeutic targets for understanding the pathological mechanisms of RA and drug development [67]. In particular, quercetin and icariin, which respectively enhance cell survival in synovial cells by targeting Caspase-8-mediated ferroptosis and pyroptosis and by inhibiting ferroptosis through the Xc-/GPX4 axis, show potential as potential therapeutic drugs for RA [68]. At the same time, total triterpenes from Euphorbia pekinensis also have a relieving effect on RA through the Nrf2/HO-1/GPX4 pathway [69]. These findings offer novel insights and tactics for the management of RA. The medicines and chemicals used for treating RA that target the ferroptosis pathway are listed in Table 1.

**Table 1:** Clinical drugs and experimental compounds have been proven to induce ferroptosis in RA.

Compound/target	Model	Dose administered	Functional mechanism	Refs.
Sulfasalazine	Fibroblast-like synoviocytes	*In vitro* concentration range 30–100 μM	Promoting ferroptosis *via* AKT-ERK1/2 and P53-SLC7A11	[70]
Semaphorin 5A	RA-SFs	*In vitro* concentration range 100–200 μM	Suppressing ferroptosis *via* PI3K-AKT-mTOR activation	[71]
NS8593	C28/I2 cells	*In vitro* concentration range 10–250 μM	Inhibiting chondrocyte ferroptosis and alleviates cartilage injury in rat adjuvant arthritis through TRPM7/HO-1 pathway	[72]
Asiatic acid	RA fibroid synovial cells; rat model of type II CIA	*In vitro* concentration range 0.5–2 μM; *In vivo* concentration range 2–4 mg/kg	Inducing ferroptosis of RA-FLS *via* Nrf2/HMOX1 pathway to relieve RA inflammation	[67]
Etanercept	Collagen-induced arthritis mouse models	*In vitro* concentration range 1–4 mM; *In vivo* concentration range 500 mg/kg	Sensitizing synovial fibroblasts to ferroptosis	[31]
Emodin	CIA rats	*In vitro* concentration range 10–40 μM	Inhibiting ferroptosis pathways and reducing MMP3/MMP13 expressions	[73]
BzATP	CIA mice	*In vitro* concentration range 10–40 μM; *In vivo* concentration range 20–40 mg/kg	Reversing ferroptosis-induced gut microbiota disorders	[74]
Anemoside B4	Mouse model of collagen-induced arthritis	*In vitro* concentration range 10–40 μM; *In vivo* concentration range 50–100 mg/kg	Suppressing ferroptosis-mediated inflammation.	[75]
Quercetin	Fibroblast-like synovial cells	The best dose is load dose 50 mg/day × 3 days, maintenance dose 20 mg/day	Caspase-8 is a key biomarker for ferroptosis and pyroptosis in RA, and quercetin is a potential therapy targeting caspase-8.	[76]
Plumbagin	Chondrocytes exposed to H_2_O_2_; rat model of unilateral anterior crossbite-induced TMJOA	*In vitro* concentration range 10–40 μM	Alleviating TMJ OA progression by inhibiting chondrocyte ferroptosis *via* MAPK pathways	[77]
Icariin	Lipopolysaccharide-induced synoviocytes	*In vitro* concentration range 0.5–2 μM; *In vivo* concentration range 2–4 mg/kg	Enhancing cell survival in LPS-induced synoviocytes by suppressing ferroptosis *via* Xc-/GPX4 axis	[68]
Mitomycin C	Fibroblast-like synoviocytes	*In vivo* concentration range 100 mg/kg	Induceing apoptosis in RA fibroblast-Like synoviocytes *via* mitochondrial pathway	[78]
Notopterygium	Assisted arthritis (AA) rats	*In vivo* concentration range 50 mg/kg	Involving NLRP3 Regulation *via* Mitochondria	[79]
Total triterpenes of Euphorbium	The rat model of RA	*In vivo* concentration range 20–100 mg/kg	Inhibiting lipid peroxidation and abnormal ferroptosis, possibly involving the Nrf2/HO-1/GPX4 pathway	[80]
Shikonin	RA FLS and adjuvant-induced arthritis (AIA) rat synovium	*In vivo* concentration range 100 mg/kg	Inducing apoptosis and autophagy *via* AMPK/mTOR/ULK-1 pathway modulation; activating ROS production, inhibiting intracellular ATP levels, glycolysis-related proteins, and the PI3K-AKT-mTOR pathway	[81,82]
Arctigenin	RA FLS	*In vitro* concentration range 10–40 μM	Inhibiting RAFLSs proliferation, inducing mitochondrial apoptosis, associated with NF-κB and Akt signaling modulation	[83]
Clematichinenoside AR	RA FLS	*In vitro* concentration range 10–50 μM	Degeneration of ROS, increase in mitochondrial membrane potential, suppression of durative JNK phosphorylation.	[84]
Amiloride	Articular chondrocytes	*In vivo* concentration range 20–50 mg/kg	enhancing anti-apoptotic ability and down-regulating pro-apoptotic factors, preserving mitochondrial function	[85]
7,3′-dimethoxy hesperetin	RA FLS and AA rats	*In vivo* concentration range 20–40 mg/kg	Caspase 3 activation	[86]
Nobiletin	RA FLS	*In vivo* concentration range 30–50 mg/kg	Suppressing IL-21/IL-21 receptor-mediated inflammatory response in	[87]
Osthole	FLS and CIA rat model	*In vitro* concentration range 10–40 μM	A potential AMPK agonist that inhibits NLRP3 inflammasome activation	[88]
7-Hydroxycoumarin	FLS and CIA rat model	*In vivo* concentration range 30–50 mg/kg	Suppression of Wnt/β-catenin signaling pathway	[89]
Leflunomide	AA rats	*In vitro* concentration range 5–10 μM; *In vivo* concentration range 20–30 mg/kg	Interferes with the metabolism of pyrimidine by inhibiting dihydroorotate dehydrogenase (DHO-DH) in mitochondria, thereby blocking T- and B cell proliferation	[90]
Resveratrol	RA-FLS and AA rats	*In vivo* concentration range 30–50 mg/kg	Activates caspase-3/89, leading to modulation of mitochondrial apoptotic machinery8	[[91], [92], [93], [94], [95], [96], [97], [98]]
Rosmarinic Acid	RA-FLS	*In vivo* concentration range 30–50 mg/kg	Mitochondrial Pathway	[99]
Methyl Jasmonate	AA rats	Fe3O4: 10 mg/kg joint cavity injection + lanzasulfonamide pyridine: 100 mg/kg peritoneal injection, combined with 808 nm laser (1 W/cm^2^, 10 min); Macrophage load: Fe3O4 100 μg/mL + lyxazole sulfapyridine 20 μM	Increasing mitochondrial isocitrate dehydrogenase activity, aiding NADPH supply for the GSH cycle, potentially contributing to partial GSH/GSSG ratio recovery	[100]
Eechinacoside	CIA rats	20–50 mg/kg joint cavity injection + 808 nm laser; 5–10 mg/kg Fe3O4 loaded anti-inflammatory drug	Nrf2/Drp1 pathway	[101]
Scopoletin	Fibroblast-like synoviocytes	IL-17 inhibitor: biological patch load 1–5 mg/kg (local slow release); ferroptosis regulator: biological patch load 5–10 mg/kg (local slow release)	A mitochondrial-dependent pathway	[102]
Andrographolide	RA-FLS	*In vitro* experiment: overexpress sEV of miR-433-3p 100 μg/mL, combined with erastin 10 μM; *In vivo* experiment: sEV 300–500 μg/kg local injection, combined with erastin 2–5 mg/kg peritoneal injection	Promotion of cytochrome C release from mitochondria and activation of caspase-3	[103]
Platycodin D	RA-FLS	*In vitro* experiments: CuSO4 50–100 μM + chloroquine 20 μM (inhibited autophagy) or rapamycin 50 nM (activated autophagy); *In vivo* experiment: CuSO4 5–10 mg/kg + chloroquine 50 mg/kg, intraperitoneal injection	Promoting apoptosis of mitochondria to inhibit activation of hedgehog pathway	[104]
Irisin	RA-FLS	*In vivo* experiment: HIF-2α inhibitor (30–50 mg/kg) + anti-VEGF antibody (5–10 mg/kg), intraperitoneal injection or joint cavity injection; *In vitro* experiment: HIF-2α inhibitor (5–10 μM) + anti-VEGF antibody (5 μg/mL), incubated for 24 h	Suppressing mitochondrial fission *via* inhibiting YAP-Drp1 signaling pathway	[105]
The Flavonoid Naringenin	CIA rats	*In vitro* concentration range 10 μM	Regulating Mitochondrial Fission	[106]
Tanshinone IIA	RA-FLS	*In vitro* concentration range 10–50 μM	Blockade of the Cell Cycle in the G2/M Phase and a Mitochondrial Pathway	[107]
α-Mangostin	RA-FLS	*In vivo* concentration range 1–5 mg/kg	Reduced mitochondrial membrane potential accompanied by cytochrome *c* accumulation in cytosol and elevated caspase-3 and caspase-9 activities	[108]
SkQ1	AA rats	*In vivo* concentration range 20–50 mg/kg	Decreasing the level of mitochondrial ROS, resulting in the suppression of the inflammatory response	[109]
Imperatorin	RA-FLS	*In vitro* concentration range Spaxacin 50 μM + rhythium nanoenzyme 100 μg/mL	Mitochondrial/caspase-mediated pathways	[110]
Cantleyoside	Human HFLS-RA cell line	*In vitro* concentration range 1–10 μM; *In vivo* concentration range 0.5–2 mg/kg	Enhancing mitochondrial dysfunction, confines inflammatory response, and promotes apoptosis *via* AMPK/Sirt1/NF-κB pathway activation	[111]
Norisoboldine	RA FLS and AA rats	*In vitro* concentration range 10 μM; *In vivo* concentration range 30–50 mg/kg	A mitochondrial pathway, possibly mediated by cytochrome C release and Bax/Bcl-2 regulation, with p53 potentially	[112]
Daphnetin	RA-SFs and CIA rats	*In vitro* concentration range 10–50 μM; *In vivo* concentration range 5–20 mg/kg	Inducing apoptosis in fibroblast-like synoviocytes from collagen-induced arthritic rats mainly *via* the mitochondrial pathway	[113]
Galangin	RA FLS and CIA rats	Dose administered	Downregulating the phosphatidylinositol 3-kinase/protein kinase B signaling pathway	[114]

## 4 NDDSs designed based on different pathways of ferroptosis

### 4.1 Drug delivery system designed based on iron ion regulation pathway

#### 4.1.1 Design of iron-based NDDSs

**Fig. 3:**
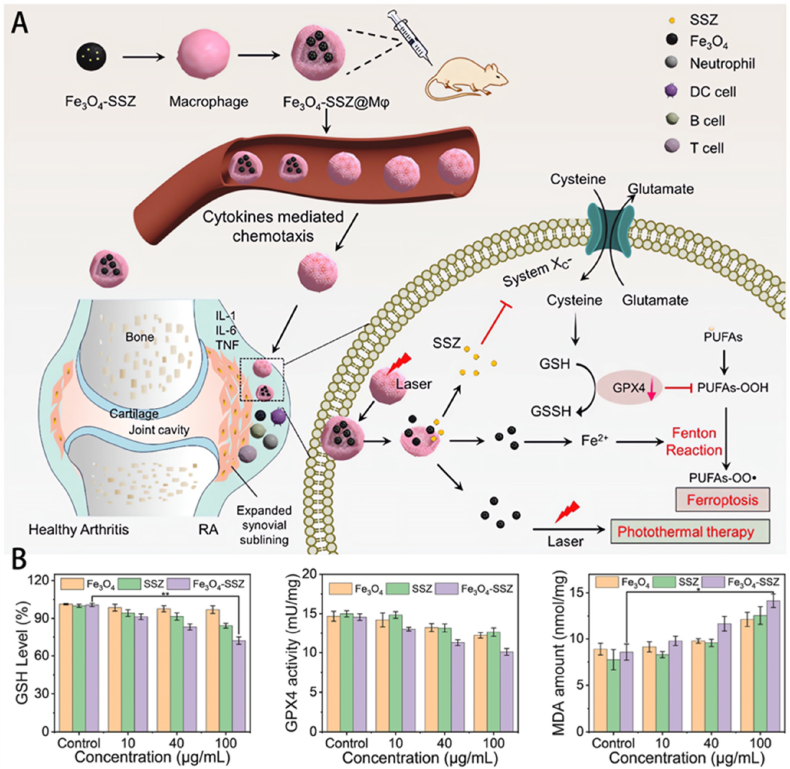
(A) Mφ-mediated drug delivery for ferroptosis and photothermal therapy of RA. (B) After exposure to varying concentrations of Fe_3_O_4_, sulfasalazine, and Fe_3_O_4_-sulfasalazine, the macrophages exhibited alterations in the levels of GSH, GPX4, and malondialdehyde activity [115]. Copyright © 2023 The Authors. Published by Elsevier Ltd. This article is available under the Creative Commons CC-BY-NC-ND license and permits non-commercial use of the work as published, without adaptation or alteration provided the work is fully attributed.

The majority of NDDSs designed to induce ferroptosis by controlling iron metabolism are mostly composed of iron, with iron oxide nanoparticles serving as the central focus of investigation. These NDDSs can serve as iron sources, releasing Fe^3+^, Fe^2+^, or iron ions, and inducing ferroptosis. Ruan et al. employed macrophages as transporters to administer Fe_3_O_4_ nanoparticles and sulfasalazine for the purpose of treating ferroptosis and conducting photothermal treatment on RA (Fig. 3) [115]. Guided by inflammatory factors, macrophages have the inherent ability to migrate to inflamed joints, allowing them to specifically deliver drugs to RA lesions. Under near-infrared light irradiation, Fe_3_O_4_ nanoparticles convert light energy into thermal energy to destroy proliferating synovial tissue. At the same time, iron ions released from Fe_3_O_4_ nanoparticles synergize with sulfasalazine to produce a synergistic effect of ferroptosis. Resident inflammatory cells and proliferating synovial tissue are effectively destroyed by the effects of ferroptosis and photothermal therapy, showing significant therapeutic effects on RA. Fe_3_O_4_ nanoparticles have demonstrated efficacy as a nanoplatform for targeted drug administration, stimulus-responsive drug release, MRI diagnostics, photothermal therapy, and magnetic hyperthermia in the treatment of RA. Fe_3_O_4_ nanoparticles possess a distinctive property known as superparamagnetism, which allows them to function as carriers for targeted medication delivery, MRI contrast agents, and transducers for thermal therapy. Moreover, under acidic conditions, ferrous ions have the ability to engage in the Fenton reaction, which produces extremely harmful hydroxyl radicals. This presents a novel approach for the therapy of RA. Fe_3_O_4_ nanoparticles have properties that make them very suitable for stimulus-responsive drug release and combination therapies, such as chemo-thermal therapy. This has the potential to result in improved therapeutic outcomes and minimized adverse effects. Fe_3_O_4_ nanoparticles possess multifunctionality, rendering them a comprehensive approach for the synergistic treatment of RA. However, it is imperative to conduct fundamental research to gain a profound understanding of the impacts of synergistic treatment and investigate its probable processes. In order to enhance the effectiveness of Fe_3_O_4_ nanoparticles as a therapy method for RA, it is necessary to create Fe_3_O_4_ nanoparticles with improved design that can increase the efficacy of magnetic heating. This will allow for the use of magnetic hyperthermia in the clinical treatment of RA. The utilization of Fe_3_O_4_ nanoparticles in the treatment of RA not only offers an efficient method for diagnosing and treating RA, but also has the potential to advance the creation of novel magnetic materials with enhanced performance capabilities [116]. The advantages of using Fe_3_O_4_ nanoparticles to treat RA through targeted hydroxyl radical release include: precise magnetic field-guided localization to inflamed joints, localized destruction of pro-inflammatory cells, immune modulation with reduced systemic side effects, and enhanced efficacy when combined with photothermal/photodynamic therapies. However, drawbacks involve potential collateral damage to healthy tissues by free radicals, risks of iron overload with prolonged use, complex and costly nanoparticle fabrication, and unresolved safety concerns such as material stability and immune responses. Further validation is required for clinical translation.

Guo et al. developed a targeted delivery system (TFSB) based on metal-organic frameworks (MOFs) [117]. This system utilizes tannic acid (TA) and Fe^3+^ self-assembly to load TNF-α siRNA, modified with bovine serum albumin (BSA), achieving dual functions of antioxidation and gene therapy. The MOF precisely targets inflamed synovial joints and M1 macrophages, releasing siRNA in acidic environments to suppress pro-inflammatory factors (*e.g.*, TNF-α, IL-6) while scavenging reactive oxygen/nitrogen species (RONS), thereby promoting macrophage polarization toward the anti-inflammatory M2 phenotype. Experiments demonstrated its ability to significantly alleviate joint damage in RA animal models and prolong drug efficacy, with advantages including high drug-loading capacity and biosafety. Future efforts should focus on optimizing MOF stability, exploring combination therapies (*e.g.*, anti-angiogenic drugs), and advancing clinical translation.

#### 4.1.2 Non-iron-based NDDSs design

The efficiency of ferroptosis triggered by the introduction of external iron is not just determined by the quantity of iron delivered, but is also impacted by the targeting properties of NDDSs and the effectiveness of cellular uptake. Given the outstanding drug-loading potential and controlled release characteristics of electrospun nanofibers, coupled with the extensive applicability of various types of stem cells, this innovative dual-layer strategy incorporating bioactive nanofibers opens up new prospects for the translational application of stem cell therapy in RA treatment. Liu et al. successfully synthesized a fully biodegradable conjugate with unique self-assembly capabilities, which can form bioactive nanoparticle micelles [118]. From a mechanistic standpoint, the micelles efficiently mitigate the inflammatory reaction of human periodontal ligament stem cells by modulating IL-17 signaling and ferroptosis pathways. Previous studies indicate that blocking IL-17 signaling can lower the production of inflammatory cytokines/chemokines, diminish the infiltration of neutrophils, and encourage the transformation of T helper cells into regulatory T cells. At the same time, the attenuation of ferroptosis pathways helps reduce the release of free iron and ROS, thereby decreasing cell death and inflammation and actively promoting bone regeneration. Therefore, as an efficient nanotherapy, bioactive nanoparticle micelles significantly enhance the function of stem cells during bone regeneration.

### 4.2 Drug delivery system designed based on lipid peroxidation pathway

Polyunsaturated fatty acid is a key substrate for lipid peroxidation during ferroptosis. Apart from the classic enzymatic Fenton reaction pathway that generates lipid peroxidation, the scientific community has made significant achievements in exploring efficient lipid peroxidation generators in recent years. These unique generators not only enhance our comprehension of Ferroptosis mechanisms but also offer inventive approaches for the management of RA. The ferroptosis of RA-FLS is induced by an inflammatory milieu that is comparable to the effects of lipopolysaccharide or erastin therapy. They can elevate intracellular levels of ROS, hence promoting the generation of lipid peroxides (lipid-ROS) from polyunsaturated fatty acids present in the cell membrane. This process disrupts the equilibrium of the lipid bilayer in the cell membrane. During the process of RA-FLS, there is an increase in the expression of endosomal protein complex required for transport-III, which is responsible for repairing cellular damage induced by ferroptosis stimulation. This repair mechanism is followed by the creation and release of small extracellular vesicles (sEV). sEV have the ability to merge with adjacent RA-FLS, resulting in an increase in the production of forkhead box-O1 and its downstream factor, vascular endothelial growth factor. This, in turn, enhances the growth of blood vessels in the synovial tissue, namely in human dermal microvascular endothelial cells, and worsens the development of arthritis. Lin et al. reveal that small extracellular vesicles (sEVs) secreted by synovial mesenchymal stem cells (SMSCs) overexpressing miR-433-3p can inhibit angiogenesis triggered by ferroptosis-associated synovial cell-derived sEVs [119]. The core mechanism lies in miR-433-3p targeting the STAT3/TLR4 signaling pathway to block pro-angiogenic factors (*e.g.*, VEGF) released by ferroptotic synovial cell sEVs. Both *in vitro* (endothelial cell migration/tube formation assays) and *in vivo* (RA animal models) experiments confirmed its inhibitory effects on pannus formation and joint inflammation. The study innovatively proposes using engineered sEVs to deliver miRNAs for dual intervention in ferroptosis and angiogenesis, offering a potential nanotherapeutic strategy for rheumatoid arthritis. However, challenges such as sEV heterogeneity screening, cross-cellular regulatory network analysis, and clinical translation safety remain to be addressed.

### 4.3 Drug delivery system designed based on antioxidant system pathways

GPX4, as a glutathione peroxidase dependent on GSH and selenium, plays a crucial role as a core inhibitor of ferroptosis in cells. By utilizing its distinctive catalytic mechanism, it has the ability to transform polyunsaturated fatty acid phospholipid hydroperoxides into equivalent phospholipid alcohols, thereby inhibiting the production of lipid hydroperoxides and consequently halting the ferroptosis process [120]. This process is vital for maintaining normal cellular physiological functions and preventing ferroptosis. The cystine/glutamate antiporter, sometimes referred to as system Xc-, is predominantly comprised of SLC7A11 at the cellular membrane level. By utilizing its unique mechanism, it enables the transfer of cystine and glutamate into and out of cells in a balanced ratio of 1:1, thus enhancing the absorption of cystine into cells. Upon entering the cell, cystine undergoes rapid reduction to cysteine, hence enhancing the production of GSH [121]. Cysteine is crucial in controlling the cellular redox state, and a decrease in its levels might result in reduced synthesis of GSH and the potential buildup of glutamate, ultimately causing the occurrence of ferroptosis. Research has demonstrated that disrupting the SLC7A11/GSH/GPX4 pathway might induce the buildup of lipid peroxides, thus initiating the ferroptosis process. Significantly, p53, a crucial gene that suppresses tumor growth, is intricately engaged in this process. The p53 protein can decrease the production of SLC7A11, which in turn hinders the absorption of cystine by system Xc-. This has an impact on the functioning of antioxidant systems like GPX4/GSH [122]. Simultaneously, p53 can also worsen the buildup of ROS and malondialdehyde, ultimately resulting in ferroptosis. However, this process can be reversed by ferroptosis inhibitors (ferostatin-1) or overexpression of SLC7A11. Further investigation has uncovered patients with RA have elevated amounts of ROS and lipid peroxidation in their serum and synovial fluid. Conversely, their blood levels of GSH and glutathione peroxidase are reduced, leading to a diminished ability to defend against oxidative stress. In lipopolysaccharide-induced synovial cells, GPX4 levels are decreased, and the classic ferroptosis pathway, the system Xc-/SLC7A11/GPX4 axis, is significantly inhibited [123].

**Fig. 4:**
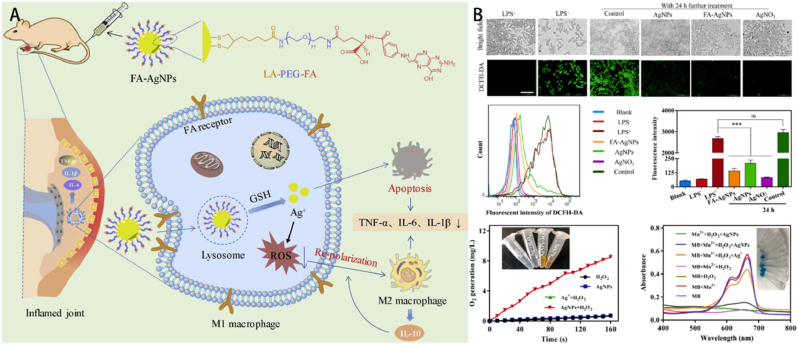
(A) Schematic depiction of the therapeutic pathway of FA-AgNPs in alleviating RA. (B) ROS scavenging capabilities [128]. © 2020 Elsevier Ltd. All rights reserved.

The GSH levels in individuals with RA are widely recognized to be greatly increased. This is strongly linked to the adaptive upregulation mechanism of GSH within cells, which aims to effectively protect cells from potential harm caused by oxidative stress. More precisely, the concentration of GSH in the cytoplasm of cells from patients with RA is unusually elevated, reaching levels 100 to 1000 times higher than the concentration of GSH in normal tissue fluid [124]. This significant increase in GSH levels not only reflects the special biochemical environment under RA pathological conditions but also provides new ideas for developing treatment strategies for RA [125]. The design of these dual-stimulus responsive nanoparticles aims to endow them with more precise regulatory capabilities, allowing for accurate modulation of drug release kinetics according to treatment needs. By cleverly combining response mechanisms to external stimuli (temperature, magnetic field, light) and internal stimuli (specific biomolecules, pH changes, redox potential), these nanoparticles can achieve intelligent drug release in complex and variable biological environments, thereby maximizing the therapeutic value of drugs during the treatment process [126]. For instance, Dang et al. effectively synthesized methotrexate stimulus carriers that are both targeted and dual-responsive for obtaining PCAC-FA carrier [127]. This was achieved by incorporating folic acid and pluronic F127 into an alginate backbone, with cystamine serving as a bridge. This engineered system demonstrates suitable particle size and excellent biocompatibility, while offering optimal release of methotrexate induced by cold temperatures. Furthermore, the deliberate accumulation of the specifically designed carriers effectively reduces the symptoms of RA by directly targeting and modifying macrophages in their original location. The solubility of methotrexate (MTX@PCAC-FA) in water is increased by a factor of 333 compared to the original methotrexate, thanks to the developed carriers. Notably, in laboratory investigations, it has been observed that the release of methotrexate from PCAC-FA nanoparticles is considerably sped up when GSH is present in the substance used for release. Moreover, in RA joint lesions, macrophages are the primary cells affected by ferroptosis. By either deleting M1 macrophages or transforming them into anti-inflammatory M2 phenotypes, it is possible to reduce synovial inflammation. Yang et al. created silver nanoparticles (AgNPs) that were modified with folate (FA-AgNPs) (Fig. 4) [128]. These nanoparticles were designed to specifically target and deliver to M1 macrophages. The treatment with FA-AgNPs resulted in a simultaneous decrease in M1 macrophages and an increase in M2 macrophages, leading to an effective treatment for RA. FA-AgNPs undergo dissolution and release of Ag when exposed to intracellular GSH, resulting in the simultaneous induction of apoptosis in M1 macrophages and scavenging of ROS. This process leads to the transformation of M1 macrophages into the M2 phenotype within inflamed synovial joints. The release of Ag in response to GSH can be explained by the affinity of Ag for GSH, which leads to strong binding between them. This binding promotes the dissolution of Ag from the surface of AgNPs.

### 4.4 Drug delivery system designed based on mitochondrial metabolic pathways

As the core site for intracellular iron utilization, mitochondria are not only a key component of cellular iron metabolism but also the primary site of ROS production. Ferroptosis, an atypical type of cellular demise, is strongly associated with the aberrant hyperpolarization of the mitochondrial membrane and the buildup of lipid peroxidation products. This underscores the pivotal involvement of mitochondria in the ferroptosis process. Further research has shown that erastin, a compound capable of inducing ferroptosis, acts by stimulating the production of ROS within mitochondria. As a result of this activation, the mitochondrial permeability transition pore opens, causing the dissipation of mitochondrial membrane potential and a significant fall in adenosine triphosphate levels. This series of events ultimately promotes the occurrence of ferroptosis, indicating that dramatic changes in mitochondrial function are a critical step in the execution of ferroptosis. It is noteworthy that cells sensitive to ferroptosis often exhibit smaller mitochondrial morphologies, which may reflect specific adaptive changes in mitochondrial structure and function in response to ferroptosis stimuli. Mitochondria have a vital function in cellular energy metabolism as the primary regulator of oxidative phosphorylation. Moreover, mitochondria play a vital role in regulating iron metabolism and maintaining balance within the body, which has significant consequences for sustaining normal physiological processes. They play an active role in the creation of iron-sulfur clusters and heme, which are crucial for numerous metabolic events in cells. Additional investigation has uncovered that the diminutive compound erastin, which induces ferroptosis, has the ability to selectively focus on the voltage-dependent anion channel located on the membrane of the mitochondria. This interaction leads to mitochondrial dysfunction, triggering a series of cascading reactions that ultimately result in ferroptosis. This finding not only enhances our comprehension of the mechanics of ferroptosis but also emphasizes the pivotal role of mitochondria in determining the destiny of cells. Moreover, the utilization of antioxidants that specifically target mitochondria can successfully prevent the onset of ferroptosis, hence strengthening the pivotal involvement of mitochondria in the process of ferroptosis triggered by cysteine deficiency. Therefore, mitochondria are not only the center of cellular energy metabolism but also a key node in regulating various cell fate decisions, including ferroptosis, providing new perspectives and potential intervention strategies for understanding resistance to RA treatment.

**Fig. 5:**
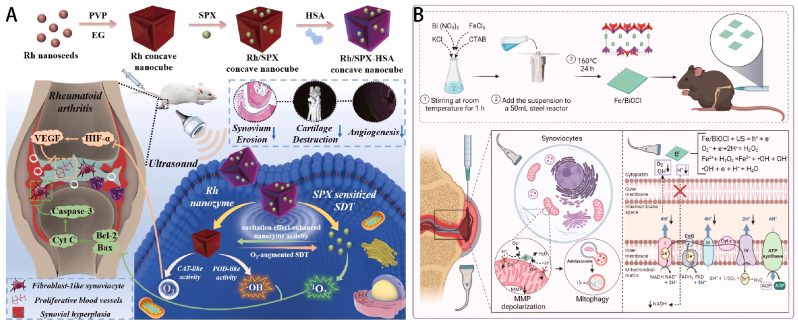
(A) Schematic depiction of Rh-SPX/HSA synthesis and its underlying therapeutic mechanisms for RA treatment [129]. Copyright © 2021 Elsevier Ltd. All rights reserved. (B) Schematic illustration of ultrasound-assisted synthesis of Fe/BiOCl nanosheets for RA treatment [131]. Copyright © 2023, American Chemical Society.

Numerous studies have been dedicated to designing NDDSs with the aim of effectively regulating mitochondrial metabolism. Li et al. have created a novel sonosensitizer called sparfloxacin (SPX) (Fig. 5A) [129]. They have included SPX into concave cubic rhodium nanozyme, along with human serum albumin. The purpose of this development is to obtain a synergistic effect in sonodynamic therapy when activated by ultrasound. SPX causes an excessive generation of ROS, which leads to malfunction of the mitochondria and thus inhibits FLS under ultrasonic circumstances. However, using concave cubic rhodium as a nanozyme with natural peroxidase and catalase-like enzyme activities not only reduces joint hypoxia resistance to angiogenesis but also greatly improves the effectiveness of sonodynamic therapy by dramatically increasing the level of ^1^O_2_. It is noteworthy that the cavitation effect of ultrasound also enhances the activity of the nanozyme, thereby achieving mutually enhanced sonodynamic therapy effects. Overall, this rhodium-based strategy achieves effective sonodynamic therapy in hypoxic microenvironments, providing broad prospects for efficient treatment of RA. Lee et al. have produced dexamethasone-carbon nanotube conjugates to enhance drug absorption and facilitate efficient intracellular drug release [130]. These conjugates enhance the process of caveolin-dependent endocytosis, leading to improved intracellular drug delivery. Additionally, they decrease the expression of major pro-inflammatory cytokines in human FLS that are activated by TNF-α at low drug doses. More precisely, dexamethasone on polyethylene glycol-coated carbon nanotubes specifically affects the mitochondria that are released from early endosomes in TNF-α-stimulated FLS. This action leads to the uptake of caveolin, restoration of mitochondrial function, and inhibition of ROS production. Current nanocatalytic therapies are limited to catalyzing the breakdown of ROS and are unable to target the underlying source of ROS generation, which is mitochondrial dysfunction. Li et al. have developed an ultrasound piezocatalytic therapy that can precisely and selectively trigger mitochondrial autophagy for the treatment of RA (Fig. 5B) [131]. The Fe/BiOCl nanosheets, which have been produced with piezoelectric properties, exhibit excellent catalytic activity in the presence of ultrasound stimulation, allowing for efficient electron generation. The electrons assimilate H^+^ ions on the external membrane of mitochondria, disrupting the provision of H^+^ ions in the mitochondrial matrix. This results in the depolarization of the mitochondrial membrane potential, which initiates the process of autophagy in mitochondria located in inflammatory areas, with the purpose of eliminating the origin of ROS regeneration. Experimental evaluations of cells and models of RA have shown that piezoelectric ultrasound catalytic treatment using Fe/BiOCl nanosheets can cure RA by stimulating mitochondrial autophagy. This finding not only elucidates the process of piezoelectric ultrasound catalytic therapy but also suggests a promising approach for utilizing piezoelectric materials in the biomedical field.

**Fig. 6:**
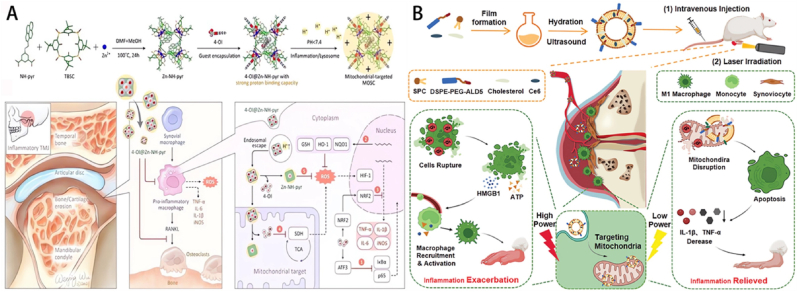
(A) A comprehensive schematic representation of the synthesis process, encapsulation technique, and therapeutic application of 4-OI@Zn-NH-pyr for amplified treatment efficacy in severe joint inflammation [132]. This is an open access article distributed under the terms of the Creative Commons Attribution License (https://creativecommons.org/licenses/by/4.0/). See http://ivyspring.com/terms for full terms and conditions. (B) A conceptual schematic depicting the mitochondrial-targeting photodynamic therapy approach for achieving a “Less-is-More” therapeutic strategy [134]. Copyright © 2024 Wiley-VCH GmbH.

Nanoparticles specifically engineered to target mitochondria can moderately boost the effectiveness of causing ferroptosis. Fan et al. created a polymer containing selenium-substituted cystamine, which functions as a carrier nanoplatform that responds to ROS (Fig. 6A) [132]. The medicinal drug used in this nanoplatform is berberine. From a mechanistic standpoint, the simultaneous use of adenosine 5′-monophosphate (AMP)-activated protein kinase (AMPK) inhibitors or palmitic acid prevented the anti-RA benefits of micelles. This suggests that micelles exert their action by specifically targeting mitochondria, limiting the formation of fat cells, and ultimately decreasing the growth of cells. ROS-responsive micelles selectively target mitochondria in inflamed tissues, leading to the elimination of afflicted cells and demonstrating a therapeutic efficacy that is tenfold greater than that of berberine. Thus, these intentionally created berberine nanoparticles that respond to ROS offer a practical approach to enhance medication accumulation in tissues injured by RA and achieve desirable treatment outcomes. In the future, the effectiveness of targeted therapy using responsive micelles can be improved by combining them with targeting antibodies, such as anti-TNF, which can facilitate binding to cells affected by RA. Furthermore, a zinc-based coordination container that has been modified with an imino-pyrene dicarboxylic acid ligand (NH-pyr) acts as a substance that can absorb protons and store electrons [133]. It has a high capacity for binding protons and can also donate and accommodate electrons in inflammatory environments. The research has been demonstrated to be an optimal vehicle for efficiently evading endo/lysosomes and specifically targeting mitochondria, as well as for proficiently scavenging ROS. The optimal proton binding capacity of Zn-NH-pyr obviates the necessity for conventional triphenylphosphonium-based modifications. Zn-NH-pyr, being an electron-rich reservoir, demonstrates excellent performance in scavenging ROS owing to its ability to donate electrons and accommodate them. One specific external itaconate, known as 4-OI, is efficiently enclosed within a coordinating container based on zinc. This container is used to create a new treatment system that works in combination to target inflammatory macrophages and osteoclasts. Studies conducted both in laboratory settings (*in vitro*) and in living organisms (*in vivo*) have demonstrated that 4-OI@Zn-NH-pyr not only inhibits the differentiation and activity of osteoclasts, but also effectively reduces joint inflammation. This is achieved by reducing excessive production of ROS and regulating the metabolism of mitochondria in synovial macrophages. This study introduces the use of the Zn-NH-pyr as a drug-carrying matrix with excellent proton acceptance and electron accommodation abilities. It opens up possibilities for developing supermolecular drug delivery systems that can specifically target subcellular locations and effectively scavenge ROS. The ultimate goal is to use these systems for the treatment of severe joint inflammation. The incorporation of photodynamic therapy treatment into mitochondrial targeting techniques for RA seeks to create a therapeutic effect characterized by the principle of “less is more”. This involves triggering larger amounts of death in pro-inflammatory cells by utilizing lower laser power. In a rat model of arthritis, chlorin 6 and mitochondrial targeting liposomes (Ce6@M-Lip) exhibited a unique therapeutic mechanism [134]. They can accumulate passively in inflamed joint sites and specifically enter pro-inflammatory macrophages. Once inside these cells, Ce6@M-Lip actively targets mitochondria, significantly enhancing mitochondrial dysfunction upon laser irradiation. This approach efficiently impairs mitochondrial activity, rendering pro-inflammatory macrophages more susceptible to the effects of photodynamic therapy, resulting in a notable rise in apoptosis. In addition, the study revealed that the exposure to high-power laser radiation can lead to the rupture of cells and the production of endogenous warning signals. These signals further recruit and activate additional macrophages, potentially exacerbating the inflammatory response. However, when exposed to low-power laser radiation, the Ce6@M-Lip compound that specifically targets mitochondria can successfully inhibit the progression of inflammation and greatly decrease the infiltration of pro-inflammatory macrophages as well as the release of pro-inflammatory cytokines (Fig. 6B). Overall, targeting mitochondria successfully coordinates therapeutic effects and inflammatory responses, achieving an effective photodynamic therapy treatment method for RA that avoids exacerbating inflammation. This discovery emphasizes the significant potential of mitochondrial targeting strategies in resolving the conflict between the effectiveness of anti-inflammatory measures and the worsening of inflammation in photodynamic therapy, suggesting the use of a “less is more” approach to achieve more therapeutic effects with reduced laser intensity.

Taking into account the unique properties of rigid and solid mesoporous silica nanoparticles (MSNs), intra-articular injection poses risks of increased infection and can compromise patient compliance. To improve the delivery of MSNs in RA treatment, there is a pressing need to develop a non-invasive and controllable drug delivery strategy that ensures effective percutaneous delivery of MSNs. Recently, Li et al. have innovatively utilized the technology of MSNs deep eutectic solutions to successfully develop a hydrogel dressing that enables non-invasive, multi-site precision-controlled treatment for RA [135]. This groundbreaking design primarily revolves around two key aspects: firstly, they meticulously crafted cerium-modified MSNs and efficiently loaded them with methotrexate, a first-line drug for RA treatment. The introduction of nano-cerium not only enhances the system's ability to scavenge ROS, thereby regulating the inflammatory microenvironment, but also promotes the transformation of pro-inflammatory M1 macrophages into anti-inflammatory M2 macrophages, achieving a dual effect of inhibiting inflammation at its source and promoting tissue repair. This strategy ingeniously combines ROS scavenging with macrophage phenotype modulation, enabling multi-point collaborative treatment of RA. Secondly, the team harnessed the unique properties of arginine and citric acid to formulate MSNs deep eutectic solutions. Here, citric acid serves as a hydrogen bond donor, modifying the surface of methotrexate, the functionalized nanoparticles were first engineered in an arginine acting as another hydrogen bond donor. When these two components are mixed in specific proportions and heated, they form deep eutectic solvents. This deep eutectic solution, through its distinctive “dragging” effect, significantly enhances the ability of MSNs to penetrate the skin's stratum corneum, enabling them to reach deeper skin layers and even enter the bloodstream. With their high specific surface area and large pore volume, MSNs act as drug reservoirs, dramatically increasing the methotrexate loading capacity in the hydrogel (up to 3 mg/mL), which then facilitates passive skin delivery through a high concentration gradient. Furthermore, the electrostatic interaction between the positive charges on the MSN-NH_2_ surface and the negative charges on skin cells not only strengthens the adhesion and contact time between nanoparticles and stratum corneum cells but also enlarges the surface area for drug release, ensuring sustained release and widespread distribution of methotrexate within the skin layers. Crucially, the permeation-enhancing effect of deep eutectic solvents further boosts the skin penetration efficiency of the drug, realizing a non-invasive and controlled transdermal drug delivery method. This innovation not only circumvents the pain, infection risks, and poor patient compliance associated with traditional repeated intra-articular injections but also significantly improves treatment safety and convenience, bringing a boon to RA patients.

### 4.5 Drug delivery system designed based on immunotherapy

Macrophages play a key role in the pathogenesis of RA, especially the polarization of M1 macrophages, which leads to excessive secretion of pro-inflammatory cytokines and ROS, which leads to ferroptosis in chondrocytes. The gelatin hydrogel prepared by Yang et al. can adhere to joint tissue *via* bioadhesion, preventing FA-PDA@Leon nanoparticles from being washed away by synovial fluid or cleared, thus prolonging their retention [136]. The Gel/FA-PDA@Leon hydrogel's bioadhesion and responsiveness to synovial fluid allow it to slowly release nanoparticles, achieving sustained drug release and a longer therapeutic effect. When combined, Leonurine and FA-PDA nanoparticles enable nanoparticles to target M1 macrophages, enhancing drug delivery efficiency. Meanwhile, the nanoparticles' antioxidative properties protect the drug, boosting anti-inflammatory effects. The Gel/FA-PDA@Leon hydrogel degrades gradually in synovial fluid, releasing the drug continuously. It suppresses ferroptosis by reducing inflammatory factors and having antioxidative components that eliminate ROS, thereby decreasing lipid peroxidation.

### 4.6 Drug delivery system designed based on other pathways

Coenzyme Q10, as a lipid-soluble antioxidant with significant biological activity, plays multiple crucial roles in biological systems [137]. Significantly, it functions as a crucial controller of ferroptosis, exerting a pivotal influence in preserving the balance of iron within cells and safeguarding against cellular harm resulting from excessive iron accumulation [138]. Coenzyme Q10 has gained significant popularity in medical research due to its expanding use, particularly in the treatment of autoimmune illnesses, where its anti-inflammatory properties have demonstrated promising potential [139]. Autoimmune disorders are a group of illnesses characterized by the immune system's aberrant attack on the body's own tissues. Coenzyme Q10 provides fresh perspectives on the treatment of these diseases due to its distinctive anti-inflammatory processes [140]. Jhun et al. conducted an innovative study where they utilized advanced nanotechnology to design lipid/gold hybrid nanoparticles encoded with coenzyme Q17 that target STAT10/Th3 [141]. The objective of this design is to specifically direct the anti-inflammatory properties of coenzyme Q10 towards cells and tissues associated with diseases. This will improve the effectiveness of the treatment and minimize any potential adverse reactions.

**Fig. 7:**
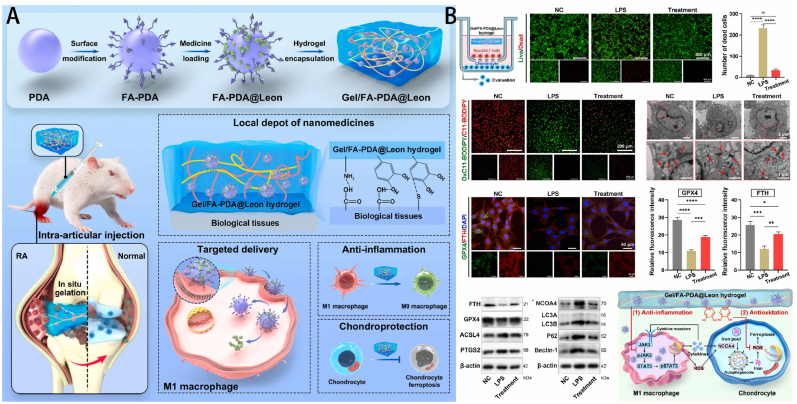
(A) Schematic diagram of the medicine-loaded nanoparticle-encapsulated hydrogel for RA treatment. (B) *In vitro* chondroprotective effect of the Gel/FA-PDA@Leon hydrogel against ferritinophagy/ferroptosis under mimicking inflammatory environment [136]. Copyright © 2024 Elsevier Ltd. All rights are reserved, including those for text and data mining, AI training, and similar technologies.

Existing drug delivery methods often require multiple administrations due to their non-targeted nature and short treatment duration, leading to suboptimal therapeutic effects and severe adverse reactions. Concurrently, the elevated concentrations of ROS within and outside cells in the synovial inflammatory milieu might lead to the deactivation of drugs. To address these issues, Yang et al. developed a local injection system that utilizes folate-functionalized polydopamine nanocarriers loaded with leonurine, which are then encapsulated into a phase-separated hydrogel composed of gelatin and poly(ethylene glycol) diacrylate to obtain the Gel/FA-PDA@Leon hydrogel (Fig. 7) [136]. This composite hydrogel exhibits multiple ideal properties as a local nanomedicine depot, including the following aspects: The nanocarriers, rich in catechol groups and folate molecules, enhance the encapsulation efficiency of the hydrophobic drug leonurine, increase the targeting ratio of M1 macrophages, protect leonurine from ROS damage, and contribute to its enhanced anti-inflammatory activity. The phase-separated hydrogel is injectable and bioadhesive, allowing long-term fixation within the joint cavity and serving as a local nanomedicine depot. The hydrogel prevents ferroptosis through a dual mechanism. Firstly, the hydrogel can limit the production of substances that cause inflammation by blocking the Janus kinase 2/signal transducer and activator of transcription 3 signaling pathway in macrophages. This, in turn, reduces the levels of nuclear receptor coactivator 4, which helps to suppress ferroptosis/iron autophagy in chondrocytes. Additionally, the hydrogel contains antioxidant catechol groups that may effectively remove excessive ROS, hence inhibiting the process of ferroptosis and reducing the level of lipid peroxidation in chondrocytes.

In addition, we summarize the NDDSs of ferroptosis for RA treatment in Table 2.

**Table 2:** Summary of NDDSs by targeting ferroptosis for RA therapy.

Treatment strategy	Drug	NDDS	Models	Effect	Ref.
Ferroptosis and photothermal therapy	Sulfasalazine, Fe_3_O_4_ NPs	Macrophage membrane	CIA mice	Fe_3_O_4_ nanoparticles convert the light into heat to damage the proliferated synovium; the iron released from Fe_3_O_4_ nanoparticles works with sulfasalazine to generate synergetic ferroptosis effect	[115]
Ferroptosis and stem cell therapy	Stem cell	Inflammation-relieving nanotherapy	Rat bone defect model	Simultaneously regulating IL-17/Ferroptosis signaling pathways	[118]
Ferroptosis and gene therapy	miR-433-3p-SMSCs-sEV	–	FLS from RA patients	Inhibiting angiogenesis induced by sEV released from synoviocytes	[119]
Ferroptosis therapy	Methotrexate	Alginate backbone was used as the bridge to connect pluronic F127 and folic acid	RA rat	On-demand release of methotrexate	[127]
Ferroptosis therapy	FA-AgNPs	–	TNF-α-FLS	In response to intracellular GSH, the release of Ag + ions occurs, leading to a synergistic effect that not only triggers apoptosis in M1 macrophages but also scavenges ROS. This dual action contributes to the polarization process, transforming M1 macrophages into the M2 phenotype within inflamed synovial joints	[130]
Ferroptosis therapy	Dexamethasone	Polyethylene glycol-carbon nanotubes	RA rat	Increasing caveolin-dependent endocytosis and recovering mitochondrial membrane potential	[130]
Ferroptosis and sonodynamic therapy	Sparfloxacin	Concave-cubic rhodium nanozyme	RA rat	Cause mitochondrial dysfunction by inducing excessive ROS production; concave-cubic rhodium was utilized as a nanozyme with endogenous peroxidase and catalase-like enzyme activities, which not only relieved the hypoxia of the joint to resist angiogenesis, but also enormously ascended the sonodynamic therapy efficacy by rising ^1^O_2_ levels	[129]
Ferroptosis and ultrasound therapy	Two-dimensional piezoelectric nanosheets	–	CIA model	The electrons that are produced are capable of efficiently attaching to and eliminating the O_2_•– and •OH radicals generated by dysfunctional mitochondria, concurrently consuming significant amounts of H^+^. This process indirectly destabilizes the MMP, triggers abnormal mitophagy, eliminates ROS, and hinders the production of inflammatory factors	[131]
Ferroptosis therapy	Berberine	Selenocystamine-based polymer	AIA rat model	Targeting mitochondrial, suppressing lipogenesis	[132]
Ferroptosis therapy	4-Octyl itaconate	Zinc-based metal-organic supercontainer	Acute TMJ arthritis model	Subcellular targeting and ROS-scavenging capacity	[133]
Ferroptosis and photodynamic Therapy	Chlorine 6	Liposomes	AIA rat model	Targeting mitochondria	[142]
Ferroptosis and immunotherapy	Methotrexate, Nanoceria-MSN, Arginine–citric acid DES	Carbomer hydrogel matrix	CIA model in rat	Initiating ROS scavenging and transformation of the macrophage phenotype	[135]
Ferroptosis therapy	Leonurine-loaded and folate-functionalized polydopamine	Gelatin-based hydrogel	CIA model in rat	Suppressing the inflammatory response by down-regulating the Janus kinase 2/signal transducer and activator of transcription 3 signaling pathway in macrophages and protect the chondrocytes from ferritinophagy/ferroptosis	
Ferroptosis therapy	CoQ10	Liposome/gold hybrid nanoparticle	CIA model in rat	*Via* STAT3/Th17 targeting	[141]

## 5 Summary and perspectives

Ferroptosis, a crucial form of regulated cell death, plays a pivotal role in both cellular homeostasis and RA pathogenesis. Recent investigations into RA disease mechanisms have revealed the clinical significance of ferroptosis regulatory factors, providing novel insights into disease etiology and potential therapeutic strategies. Our comprehensive analysis of accumulated evidence demonstrates that iron accumulation, lipid peroxidation, and inflammatory responses in RA-affected tissues collectively establish ferroptosis as a key contributor to disease progression. These findings position ferroptosis as a common pathological mechanism underlying malignancies, autoimmune diseases, and neurological disorders, while highlighting its particular relevance to RA. Emerging evidence indicates that ferroptosis resistance in FLSs may drive synovial hyperplasia, offering new perspectives on RA pathology. Notably, conventional DMARDs including methotrexate, sulfasalazine, hydroxychloroquine, and cyclophosphamide demonstrate pro-ferroptotic effects, establishing a pharmacological basis for ferroptosis induction as a joint-protective strategy. Furthermore, bioactive compounds derived from traditional Chinese medicine - including resveratrol, quercetin, and baicalein - show promising ferroptosis-inhibitory properties, representing valuable candidates for novel RA therapeutics development.

(1) Challenges and advances in RA treatment strategies

The treatment of rheumatoid arthritis (RA) faces dual challenges. (Ⅰ) Ferroptosis is closely linked to pro-inflammatory responses, iron overload, and ROS-mediated cartilage/bone damage. Overcoming ferroptosis-induced inflammatory responses primarily requires in-depth understanding of ferroptosis mechanisms—ferroptosis involves multiple signaling pathways and molecules, necessitating further research into its disease-specific mechanisms to develop precise therapeutic targets [143]. Additionally, more effective ferroptosis modulators must be developed—current ferroptosis inhibitors have limited efficacy and safety, demanding the creation of more potent and specific agents [144,145]. (Ⅱ) Traditional therapies like nonsteroidal anti-inflammatory drugs (NSAIDs), steroids, and disease-modifying antirheumatic drugs (DMARDs), despite long-term use, carry severe side effects that restrict their prolonged application. Consequently, intra-articular injections of free antirheumatic drugs and their nanoformulations have emerged as innovative strategies to enhance drug bioavailability, prolong joint retention, and improve efficacy while reducing toxicity. Meanwhile, ferroptosis-targeted therapies based on its metabolic connections are becoming a research frontier. For example, modulating autophagy-related protein BECN1 to inhibit system Xc^−^ function can promote ferroptosis, while GPX4 inhibitors like RSL3 and ML162 exacerbate lipid peroxidation to induce ferroptosis, offering new directions for RA drug development. Furthermore, studies reveal that M2 macrophages in iron-rich RA synovial environments are more susceptible to ferroptosis. Ferroptotic M2 macrophages release high mobility group box 1 (HMGB1), which activates the TLR4/STAT3 signaling pathway in M1 macrophages, exacerbating inflammation. Early administration of the ferroptosis inhibitor liproxstatin-1 or upregulating GPX4 expression in M2 macrophages effectively alleviates joint inflammation and damage, highlighting the therapeutic potential of targeting ferroptosis-related pathways (*e.g.*, HMGB1/TLR4/STAT3) in RA. These advances open new avenues for precision RA treatment *via* NDDSs and metabolic regulation [57].

(2) Future directions in RA nanotherapy

Although targeting ferroptosis offers novel therapeutic avenues for rheumatoid arthritis (RA), its clinical translation faces multiple challenges (Ⅰ) Toxicity Risks: Combination therapies (*e.g.*, ferroptosis inducers + apoptosis inhibitors): may cause metabolic interference or immune imbalance, requiring optimization of sequential dosing strategies through organoid models and single-cell sequencing. (Ⅱ) Delivery Efficiency Bottlenecks: ROS/pH-responsive carriers (*e.g.*, MOFs) are susceptible to heterogeneity in inflammatory microenvironments. Penetration efficacy can be enhanced by integrating bioinspired carriers (*e.g.*, macrophage membrane-coated nanoparticles) or ultrasound/magnetic navigation technologies. (Ⅲ) Clinical Translation Barriers: These include a lack of human-specific safety data, difficulties in scaling up nanocarrier production, and the inability of current biomarkers (*e.g.*, sTfR, MDA) to enable dynamic efficacy assessment. Future research focuses on: (Ⅰ) Novel NDDS designs: Targeting newly identified pathways (*e.g.*, iron metabolism, M1/M2 macrophage balance) and utilizing iron-based nanomaterials (iron-platinum nanoparticles, metal-organic frameworks). (Ⅱ) Advanced delivery systems: Thermally responsive hydrogels to prevent drug leakage and enhance synovial viscoelasticity, or hybrid systems integrating nanoparticles with injectable gels. (Ⅲ) Multi-mechanism therapies: By synergistically targeting multiple cell death pathways, combining ferroptosis inducers (*e.g.*, erastin) with modulators of apoptosis (*e.g.*, BCL-2 inhibitors), necroptosis (*e.g.*, RIPK1 inhibitors), or autophagy (*e.g.*, AMPK activators), this approach leverages cross-regulatory mechanisms to enhance anti-inflammatory and anti-synovial hyperplastic effects. For example, the combination of erastin (a ferroptosis inducer) and z-VAD (an apoptosis inhibitor, pan-caspase inhibitor) selectively eliminates pro-inflammatory synovial cells while preserving healthy tissue, offering a dual-pathway strategy to disrupt RA progression.

## CRediT authorship contribution statement

**Xiaolin Dai:** Writing – original draft, Software, Resources, Methodology, Investigation, Formal analysis, Data curation. **Yu Zheng:** Validation, Supervision, Resources, Methodology, Investigation. **Jianrong Cui:** Validation, Supervision, Resources, Methodology, Investigation. **Yuqi Zeng:** Validation, Supervision, Software, Resources, Project administration, Methodology. **Bo Yang:** Writing – review & editing, Funding acquisition, Formal analysis, Data curation, Conceptualization. **Zhanlin Zhang:** Writing – review & editing, Validation, Supervision, Investigation, Funding acquisition, Formal analysis, Data curation, Conceptualization.

## Declaration of competing interest

The authors declare that they have no known competing financial interests or personal relationships that could have appeared to influence the work reported in this paper.

## Data Availability

Data will be made available on request.
